# An efficient Planet Optimization Algorithm for solving engineering problems

**DOI:** 10.1038/s41598-022-12030-w

**Published:** 2022-05-19

**Authors:** Thanh Sang-To, Minh Hoang-Le, Magd Abdel Wahab, Thanh Cuong-Le

**Affiliations:** 1 Laboratory Soete, Department of Electromechanical, Systems and Metal Engineering, Ghent University, Technologiepark Zwijnaarde 903, 9052 Zwijnaarde, Belgium; 2 Faculty of Civil Engineering, Ho Chi Minh City Open University, Ho Chi Minh City, Vietnam; 3 Faculty of Mechanical - Electrical and Computer Engineering, School of Engineering and Technology, Van Lang University, Ho Chi Minh City, Vietnam

**Keywords:** Mechanical engineering, Computational science

## Abstract

In this study, a meta-heuristic algorithm, named The Planet Optimization Algorithm (POA), inspired by Newton's gravitational law is proposed. POA simulates the motion of planets in the solar system. The Sun plays the key role in the algorithm as at the heart of search space. Two main phases, local and global search, are adopted for increasing accuracy and expanding searching space simultaneously. A Gauss distribution function is employed as a technique to enhance the accuracy of this algorithm. POA is evaluated using 23 well-known test functions, 38 IEEE CEC benchmark test functions (CEC 2017, CEC 2019) and three real engineering problems. The statistical results of the benchmark functions show that POA can provide very competitive and promising results. Not only does POA require a relatively short computational time for solving problems, but also it shows superior accuracy in terms of exploiting the optimum.

## Introduction

In recent years, many nature-inspired optimization algorithms have been proposed. Some of swarm-inspired algorithms are appreciated such as Particle Swarm Optimization algorithm (PSO)^1^, Firefly Algorithm (FA)^2^, Dragonfly Algorithm (DA)^3^, Whale Optimization Algorithm (WOA)^4^, Grey Wolf Optimizer (GWO)^5^, Monarch Butterfly Optimization (MBO)^6^, Earthworm Optimization Algorithm (EWA)^7^, elephant herding optimization (EHO)^8^, moth search (MS) algorithm^9^, Slime Mould Algorithm (SMA)^10^, Colony Predation Algorithm (CPA)^10^ and Harris Hawks Optimization (HHO)^11^. Besides, quite a number of physics-inspired algorithm simulated physical laws in the universe or nature, such as Curved Space Optimization (CSO)^12^, Water Wave Optimization (WWO)^13^, etc. Moreover, some algorithms based on the mathematical foundations are also creative approaches, e.g. Runge Kutta optimizer (RUN)^14^.

On the other hand, some algorithms simulate human behavior such as Teaching–Learning-Based Optimization (TLBO)^15^, and Human Behavior-Based Optimization (HBBO)^16^. Meanwhile, Genetic Algorithm (GA)^17^ is inspired by evolution, and achieves a lot of success in solving optimization problems in many fields. With the growing popularity of GA, many evolutions-based algorithms are proposed in the literature, including Evolutionary Programming (EP)^18^, and Evolutionary Strategies (ES)^19^.

Nowadays, metaheuristic algorithms become an essential tool for solving complex optimization problems in various fields. Many researchers applied such algorithms to make an effort to deal with difficult issues in biology^20^, economics^21^, engineering^22,23^, etc. Therefore, constructing new algorithms to meet such complex requirements has a significant merit.

In this study, a strong algorithm is constructed for solving local and global optimization problems. The idea comes from the natural motion of planets in our solar system and the interplanetary interactions throughout their lifecycle. Newton's law of gravity reflects the gravitational interaction of the Sun with planets orbiting to find the optimized position through individual planets characteristics. These planets characteristics are their masses and distances.

In this paper, we propose an optimization algorithm using Newton's law of universal gravitation as the basis for its development. In this algorithm, a number of pre-eminent features are considered, such as local search, global search, to increase the ability for finding the exact solutions built into simulating the planets’ movement in the universe.

This research paper is structured into several sections as follows. In the next section, the construction of a meta-heuristic algorithm is presented. The structural POA is simulated based on Newton's law of universal gravitation and astronomical phenomena. Then, a wide range of applications of various benchmark problems is used to demonstrate how effective POA is. At the same time, we present the applications of the POA to real engineering problems. Finally, based on the results presented, the last section reports the conclusions.

## The Planet Optimization Algorithm (POA)

Physics is a fundamental science whose laws governs everything from the tiniest object electrons, neutrons, or protons to extremely massive stars or galaxies (about a hundred thousand light-years across). The laws of physics are widely applied in everyday life from transportation to medicine, from agriculture to industry, etc. In science, it is also the foundation for many other sciences such as chemistry, biology, even math. In the field of artificial intelligence (AI), the laws of physics are the inspiration for many optimization algorithms. In the study, we also present an algorithm based on such a physical law.

### Inspiration

Inspired by the laws of the motion in the universe, an algorithm is proposed from the interaction of mutual gravitational between the planets. Specifically, this optimization algorithm simulates the universal gravitation laws of Isaac Newton. The core of this algorithm is given as follows:

1 $$\left| {\mathop{F}\limits^{\rightharpoonup} } \right| = G \times m_{1} \times m_{2} /R^{2}$$

where $\overrightarrow {F}$: The gravitational force acting between two planets; $G$ : The gravitational constant; $R$ : The distance between two planets; $m_{1} ,m_{2}$: The mass of the two planets.

**Figure 1:**
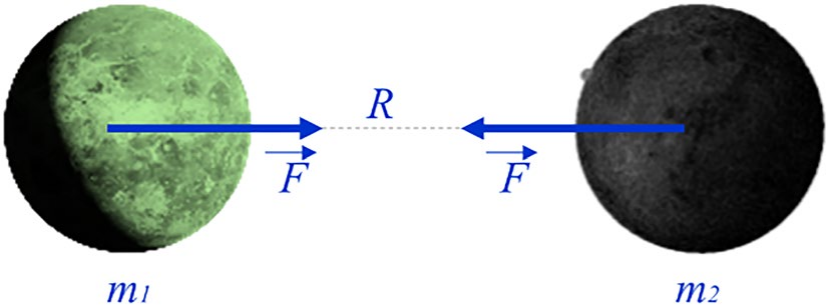
The force *F* acting between two planets.

The gravitation of a two-planet (as shown in Fig. 1) is dependent on as Eq. (1). However, in this study, we find that the value of force $\overrightarrow {F}$ will give less effective results than when using the moment $(M)$ as a parameter in the search process of the algorithm.

2 $$\left| {\overrightarrow {M} } \right| = \left| {\overrightarrow {F} } \right| \times R$$

### The planet optimization algorithm

The universe is infinitely big and has no boundary, and it is a giant space that is filled with galaxies, stars, planets, and many and many interesting astrophysical objects. For simplicity and ease of visualization, we use the solar system to make representation for this algorithm simulation.

**Figure 2:**
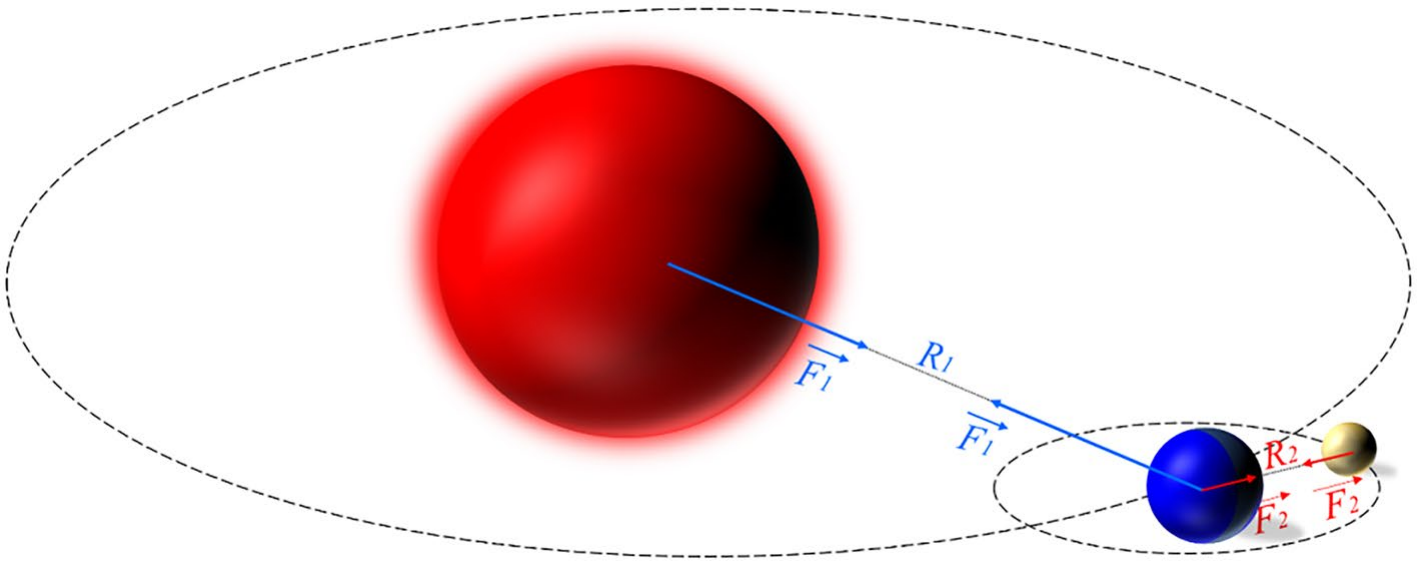
The gravitational force acting between planets.

**Figure 3:**
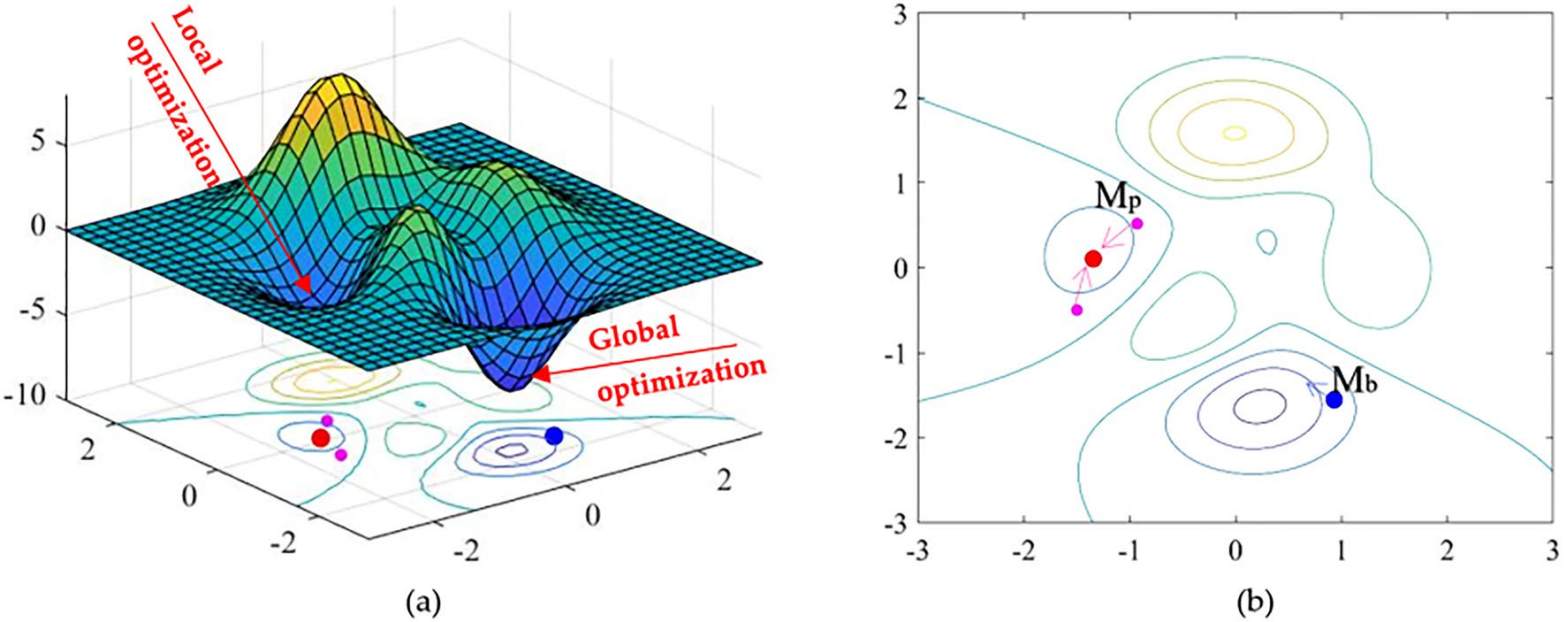
Local and global optimization: (**a**) 3D view; (**b**) plane.

First of all, a system that consists of the Sun, the Earth and the Moon (as shown in Fig. 2) is considered in this case. Of course, everybody understands that the Sun maintains its gravitation to keep the Earth moving around it. Interestingly, the mass of the Sun is 330,000 times higher than that of the Earth. However, the Earth also creates a gravitational force large enough to keep the Moon in orbit around the Earth. This demonstrates that two factors influence the motion of a planet, not only the mass but also the distance between the two planets. An algorithm simulating the law of universal gravitation is, therefore, presented as follows:

- The Sun will act as the best solution. In the search space, it will have the greatest mass, which means it will have a greater gravitational moment for the planets around and near it.
- Between the Sun other planets, there is a gravitational attraction moment between each other. However, this moment depends on the mass as well as the distance between these two objectives. This means that, although the Sun has the largest mass compared to other planets, its moment on the too distant planets is negligible. This helps the algorithm to avoid local optimization as illustrated in Fig. 3.

In the *t*th iteration, the mass of red planet (see Fig. 3) is the biggest, so it represents the Sun. As the pink planets are close to the Sun, they will move to the location of the Sun because of a gravitational attraction moment $(M_{p}^{t} )$ between the Sun and the planets.

Nevertheless, the red planet (or the Sun) in the *t*th iteration does not have the desired position that we are looking for, i.e. a minimum optimum. In other words, if all planets move to the red planet, the algorithm is stuck in the local space. In contrast, the blue planet is a potential location and far from the Sun. The interaction of the Sun with the blue planet $(M_{b}^{t} )$ is small, because it is far from the Sun in the *t*th iteration. Thus, it is quite free for the blue planet to search a better location in the next iterations.

The main core of the algorithm is based on the above 2 principles. Besides, the Sun is the true target of searching, and of course we don’t have its exact location. In this case, the planet with the highest mass in the *t*th iteration would act as the Sun at the same time.

The implementation of the algorithm is as follows:

#### Stage 1: the best start

Ideally, a good algorithm is the one in which the final best solution should be independent of the initial positions. Nevertheless, the reality is exactly the opposite for almost all stochastic algorithms. If the objective region is hilly and the global optimum is located in an isolated minor area, an initial population has an important role. If an initial random population does not create any solution in the vicinity of the global search level of the original population, the probability that the population concentrates on true optimum can be very low.

In contrast, with building initial solutions near the global optimal position, the probability of the convergence of the population to the optimal location is very high. Globalization is indeed very high, and consequently, population initialization plays a vital role. Ideally, the initiation should use the critical sampling method, such as techniques applied to the Monte Carlo method in order to sample the solutions for an objective context. This, however, requests enough intellect of the problem and cannot be satisfied for most algorithms.

Similar to choosing initial population, choosing the best solution in the original population to the role of the Sun with respect to all the other planets moving to the position is important. This selection will determine the convergence speed as well as the accuracy of the algorithm in the future.

Therefore, the algorithm's first step is to find an effective solution to play a role of the best solution to increase the convergence and accuracy of the search problem in the first iterations.

#### Stages 2: M factor

3 $$M = \left| {\mathop{F}\limits^{\rightharpoonup} } \right|R_{{{\text{ij}}}}^{{}} = G\frac{{m_{i} m_{j} }}{{R_{{{\text{ij}}}}^{2} }} \times R_{{{\text{ij}}}}^{{}}$$

In Eq. (3), the following parameters are defined:

- The mass of the planets:
  4 $$m_{i} ,m_{j} = \frac{1}{{a^{{obj_{i,j} /\alpha }} }}$$
  where $a = 2$ is a constant parameter, and $\alpha = \left| {\max (obj) - obj_{sun} } \right|$ . This means that if the objective function value of a planet is smaller, the mass of this planet is larger. $obj_{i,j} ,\max (obj),obj_{sun}$ are the values of objective function of the *i*th or *j*th planet, the worst planet and the Sun, respectively.
- The distance between any 2 objects *i* and *j* with “*Dim*” as dimensions, Cartesian distance, is calculated by Eq. (5):
  5 $$R_{{{\text{ij}}}} = \left\| {X_{i}^{t} - X_{j}^{t} } \right\| = \sqrt {\sum\limits_{k = 1}^{Dim} {\left( {X_{i}^{t} - X_{j}^{t} } \right)^{2} } }$$
- *G* is a parameter, and it is equal to unity in this algorithm.

#### Stage 3: Global search

From the above, a formula built to simulate global search is indicated by Eq. (6)

6 $$\overrightarrow {{X_{i}^{t + 1} }} = \overrightarrow {{X_{i}^{t} }} + b \times \beta \times r_{1} \times \left( {\overrightarrow {{X_{Sun}^{t} }} - \overrightarrow {{X_{i}^{t} }} } \right)$$

The lefthand side of the formula illustrates the current position of a planet *i*th in the (*t* + 1) iteration, while the righthand side consists of the main elements as follows:

- $\overrightarrow {{X_{i}^{t} }}$ is the current position of a planet *i*th in the iteration *t*th.
- $\beta = M_{i}^{t} /M_{{_{\max } }}^{t}$,$r_{1} = rand(0,1),b$ is a constant parameter.
- $\overrightarrow {{X_{Sun}^{t} }}$ is the current position of the Sun in the iteration *t*th.

where $\beta$ is a coefficient that depends on *M*, as shown in Eq. (3), in which $M_{i}^{t}$ is the Sun's gravity on a planet *i*th at *t* iteration, and $M_{\max }^{t}$ is the value of $\max (M_{i}^{t} )$ at *t* iteration. Therefore, the $\beta$ coefficient contains values in the interval (0, 1).

#### Stage 4: Local search

In the search process, the true location is always the desired target to be found. However, this goal will be difficult or easy to achieve in this process depending on the complexity of the problem. In most cases, it is only possible to find an approximate value that fits the original requirement. That is to say, the true Sun location yet is in the space between the found solutions.

Interestingly, although Jupiter is the most massive planet in the solar system, Mercury is the planet, for which its location is the closest the Sun. It means that the best solution position to true Sun location at the *t* iteration may not be closer than the location of some other solutions to the true location of the Sun.

When the distance between the Sun and planets is small, the local search process is run. As mentioned above, the planet with the biggest mass will operate as the Sun, and in that case, it is Jupiter. Planets near the Sun will go to the location of the Sun. In other words, the planets move a small distance between it and the Sun at *t* iteration instead of going straight towards the Sun. The aim of this step is to increase accuracy in a narrow area of search space. Eq. (7) indicates the process for local search as follows:

7 $$\overrightarrow {{X_{i}^{t + 1} }} = \overrightarrow {{X_{i}^{t} }} + c \times r_{1} \times \left( {r_{2} \times \overrightarrow {{X_{sun}^{t} }} - \overrightarrow {{X_{i}^{t} }} } \right)$$

where $c = c_{0} - t/T$, *t* is the *t*th iteration, *T* is the maximum number of iterations, and *c*_0_ = 2. $r_{2}$ is Gauss distribution function illustrated by Eq. (8).

8 $$f(x,\mu ,\sigma ) = \frac{1}{{\sigma \sqrt {2\pi } }}\exp \left( { - \frac{{\left( {x - \sigma } \right)^{2} }}{{2\sigma^{2} }}} \right)$$

**Figure 4:**
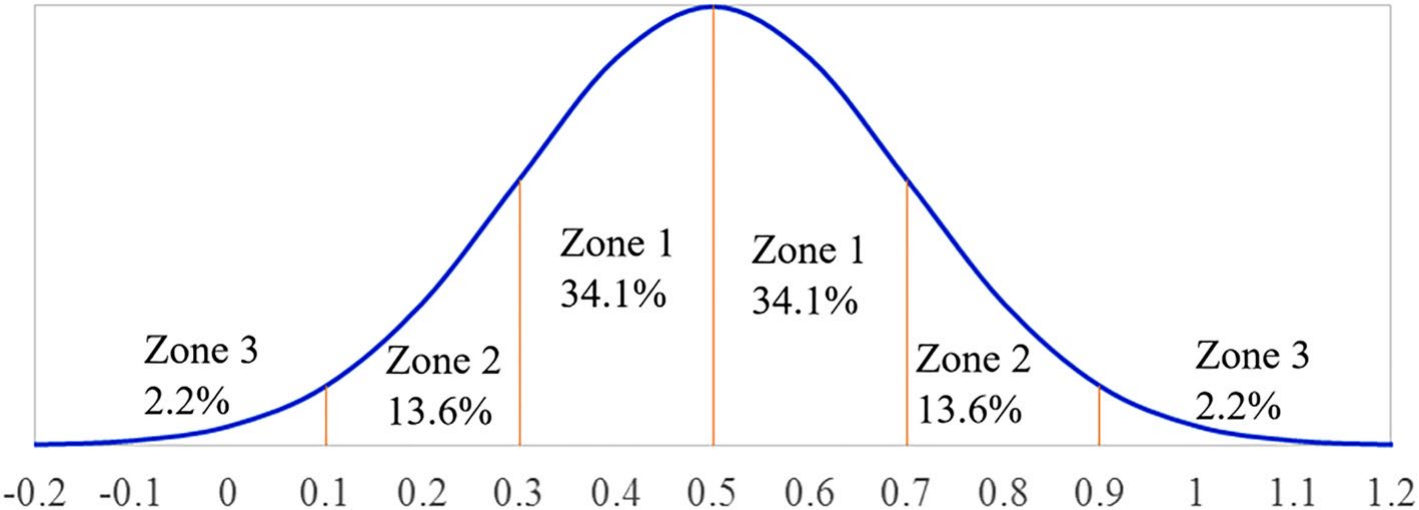
Gauss distribution.

Many evolutionary algorithms are also randomized by applying common stochastic processes such as power-law distribution and Lévy distribution. However, Gaussian distribution or normal distribution is the most popular since the large number of physical variables (see Fig. 4), including light intensity, errors/uncertainty in measurements, and many other processes, obey this distribution.

The coefficient $r_{2}$ is the Gaussian distribution with mean value $\mu = 0.5$ and standard deviation $\sigma = 0.2$. It means that 68.2% of $r_{2}$ is in zone 1 about $(\mu - \sigma ) = 0.3$ to $(\mu + \sigma ) = 0.7$, and 27.2% of its values is in zone 2 from $(\mu \pm 2\sigma )$ to $(\mu \pm \sigma )$. In other words, POA will move to around the Sun without ignoring potential solutions in local search.

Exploitation employs any data obtained from the issue of interest to create new solutions, which are better than existing solutions. This process and information (for instance gradient), however, are normally local. Therefore, this search procedure is local. The result of search process typically leads to high convergence rates, and it is the strong point of exploitation (or local search). Nevertheless, the weakness of local search is that normally it gets stuck in a local mode.

In contrast, exploration is able to effectively explore the search space, and it typically creates many diverse solutions far from the current solutions. Thus, exploration (or global search) is normally on a global scale. The great strength of global search is that it rarely gets stuck in a local space. The weakness of the global search, however, is slow convergence rates. Besides, in many cases, it wastes effort and time since a lot of new solutions can be far from the global solution.

**Figure 5:**
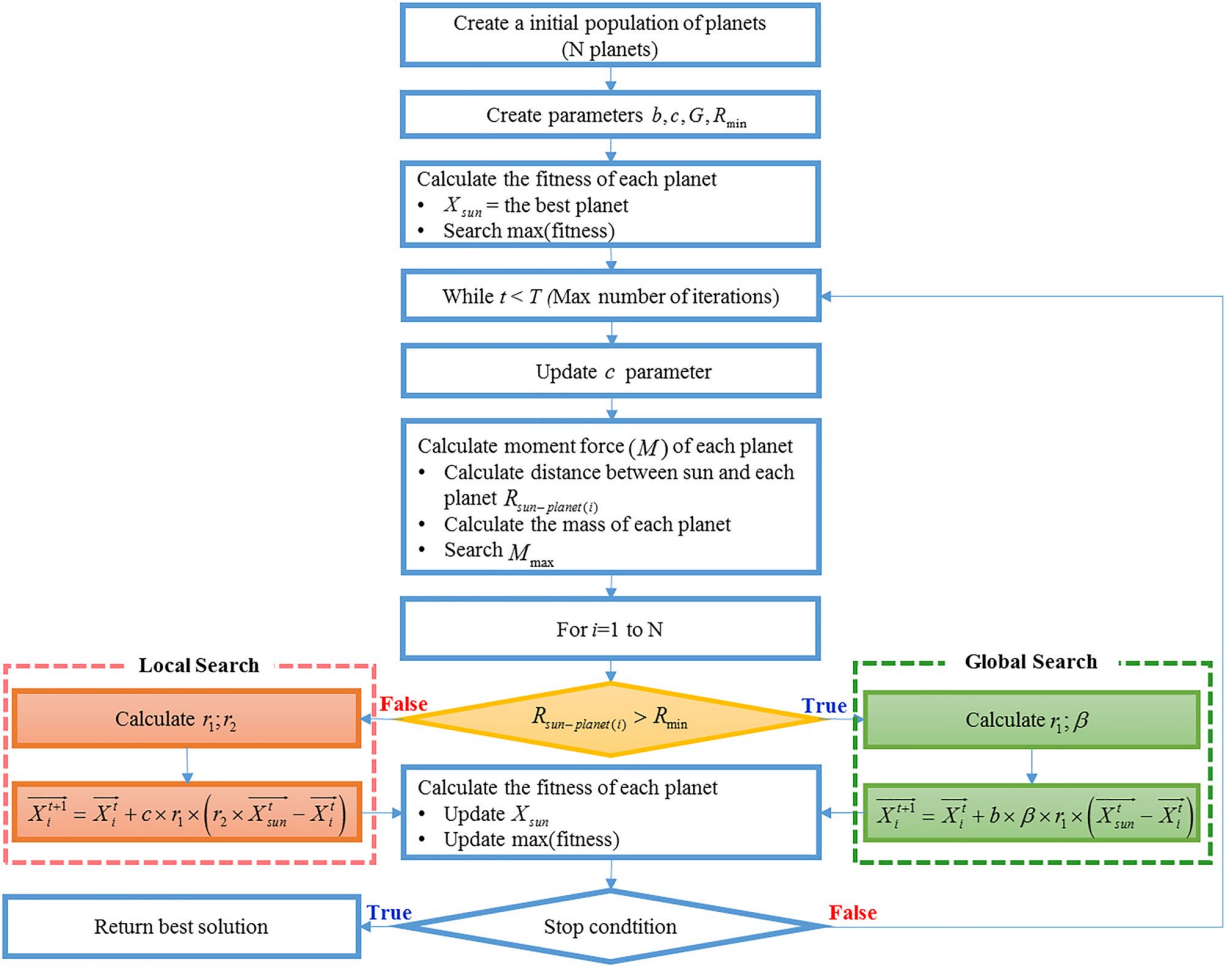
Flow chart of the proposed POA.

Figure 5 shows the operation of this algorithm, in which two local and global search processes are governed by the distance parameter *R*_min_. This means that a planet far away from the Sun will be moved depending on Newton law. In contrast, for planets very close to the Sun, the effect of the newton force is so great. They are only moving, therefore, around the Sun. A planet, which is close to the Sun, will support the Sun in exploring a local search space, as shown in Eq. (7), while the motion of distant planets from the Sun is less affected by this star at the same time. It means they have a chance to find new potential stars. Search local and global spaces runs simultaneously. This guarantees the enhancement of the accuracy of the search process, but this algorithm does not miss the potential locations.

The parameter *R*_min_ must satisfy the following two conditions:

- If the *R*_min_ is too large, the algorithm will focus on local search in the first iterations. Therefore, the probability of finding a potential location far away from the present one is difficult.
- In contrast, if *R*_min_ is too small, the algorithm focuses on global search. In other words, the exploration of POA in the zone around the Sun is not thorough. Consequently, the best value of the search process may not satisfy the condition.
  9 $$R_{\min } = \left( {\sum\limits_{1}^{Dim} {(up_{i} - low_{i} )^{2} } } \right)/R_{0}$$

In this study, *R*_min_ is chosen by dividing the search space into 1000 (*R*_0_ = 1000) zones. Where ‘low’ and ‘up’ are lower and upper bounds of each problem, respectively. With an explicit structure consisting of 2 local and global search processes, POA has satisfied the above two issues and promises to be effective and saving of time in solving complex problems.

## Results and discussion

In this section, POA is compared with a series of algorithms using well-known problems. The investigations run on the operating system of Windows 11th Gen Intel(R) Core(TM) i7-1185G7 @ 3.00 GHz 1.80 GHz with RAM 16.0 GB.

### Experimental results using classical benchmark functions

In this subsection, POA is employed to handle a wide range of applications of various benchmark problems. A set of mathematical functions with known global optima is commonly employed to validate the effectiveness of the algorithms. The same process is also followed, and a set including 23 benchmark functions in the literature as test beds are employed for this comparison^24–26^. These test functions consist of 3-group, namely unimodal (F1–F7), multi-modal (F8–F13), and fixed-dimension (F14–F23) multimodal benchmark functions. POA is compared with seven algorithms, namely PSO^1^, GWO^5^, GSA^27^, FA^2^ and ASO^28^, HHO^11^, HSG^29^ on a set of 23 benchmark functions as shown in Table 1.

**Table 1:** Classical benchmark functions.

Function (F_i_)	Range	Dim	Min_F_
$F_{1} = \sum\limits_{i = 1}^{n} {(x_{i}^{{}} )^{2} }$	[−100,100]	30	0
$F_{2} = \sum\limits_{i = 1}^{n} {\left| {x_{i}^{{}} } \right| + \prod\limits_{i = 1}^{n} {\left| {x_{i}^{{}} } \right|} }$	[−10,10]	30	0
$F_{3} = \sum\limits_{i = 1}^{n} {\left( {\sum\limits_{j - 1}^{i} {x_{j}^{{}} } } \right)}^{2}$	[−100,100]	30	0
$F_{4} = \max_{i} \left\{ {\left| {x_{i} } \right|,1 \le i \le n} \right\}$	[−100,100]	30	0
$F_{5} = \sum\limits_{i = 1}^{n - 1} {\left( {100(x_{i + 1}^{{}} - x_{i}^{2} )^{2} + \left( {x_{i} - 1} \right)^{2} } \right)}$	[−30,30]	30	0
$F_{6} = \sum\limits_{i = 1}^{n} {(x_{i}^{{}} + 0.5)^{2} }$	[−100,100]	30	0
$F_{7} = \sum\limits_{i = 1}^{n} {ix_{i}^{4} + rand\left[ {0,1} \right)}$	[−1.28,1.28]	30	0
$F_{8} = \sum\limits_{i = 1}^{n} { - x_{i} \sin \sqrt {\left| {x_{i}^{{}} } \right|} }$	[−500,500]	30	−418.9829 × Dim
$F_{9} = \sum\limits_{i = 1}^{n} {(x_{i}^{2} - 10\cos (2\pi x_{i} ) + 10)^{2} }$	[−5.12,5.12]	30	0
$F_{10} = - 20\exp \left( { - 0.2\sqrt {\frac{1}{n}\sum\limits_{i = 1}^{n} {x_{i}^{2} } } } \right) - \exp \left( {\frac{1}{n}\sum\limits_{i = 1}^{n} {\cos (2\pi x_{i} )} } \right) + 20 + e$	[−32,32]	30	0
$F_{11} = \frac{1}{4000}\sum\limits_{i = 1}^{n} {x_{i}^{2} } - \prod\limits_{i = 1}^{n} {\cos \left( {\frac{{x_{i} }}{\sqrt i }} \right)} + 1$	[−600,600]	30	0
$$\begin{gathered} F_{12} = \frac{\pi }{n}\left\{ {10\sin (\pi y_{1} ) + \sum\limits_{i = 1}^{n - 1} {\left( {y_{i} - 1} \right)^{2} \left[ {1 + 10\sin^{2} (\pi y_{i + 1} )} \right]} + \left( {y_{n} - 1} \right)^{2} } \right\} + \sum\limits_{i = 1}^{n} {u(x_{i} ,10,100,4)} \hfill \\ where:y_{i} = 1 + \frac{{(x_{i} + 1)}}{4} \hfill \\ \end{gathered}$$	[−50,50]	30	0
$F_{13} = 0.1\{ \sin^{2} (3\pi x_{1} ) + \sum\limits_{i = 1}^{n} {\left( {x_{i} - 1} \right)^{2} \left[ {1 + 10\sin^{2} (3\pi x_{i} + 1)} \right]} + \left( {x_{n} - 1} \right)^{2} {[}1 + \sin^{2} (2\pi x_{n} )]\} + \sum\limits_{i = 1}^{n} {u(x_{i} ,5,100,4)}$	[−50,50]	30	0
$F_{14} = \left( {\frac{1}{500} + \sum\limits_{j = 1}^{25} {\frac{1}{{j + \sum\nolimits_{i = 1}^{2} {(x_{i} - a_{ij} )^{6} } }}} } \right)^{ - 1}$	[−65,65]	2	1
$F_{15} = \sum\limits_{i = 1}^{11} {\left[ {a_{i} - \frac{{x_{i} (b_{i}^{2} + b_{i} x_{2} }}{{b_{i}^{2} + b_{i} x_{3} + x_{4} }}} \right]}^{2}$	[−5,5]	4	0.00030
$F_{16} = 4x_{1}^{2} - 2.1x_{1}^{4} + \frac{1}{3}x_{1}^{6} + x_{1} x_{2} - 4x_{2}^{2} + 4x_{2}^{4}$	[−5,5]	2	−1.0316
$F_{17} = \left( {x_{2}^{{}} - \frac{5.1}{{4\pi }}x_{1}^{2} + \frac{5}{\pi }x_{1}^{{}} - 6} \right) + 10\left( {1 - \frac{1}{8\pi }} \right)\cos x_{1} + 10$	[−5,5]	2	0.398
$$\begin{gathered} F_{18} = \left[ {1 + \left( {x_{1} + x_{2} + 1} \right)^{2} \left( {19 - 14x_{1} + 3x_{1}^{2} - 14x_{2} + 6x_{1} x_{2} + 3x_{2}^{2} } \right)} \right] \times \hfill \\ \times \left[ {30 + \left( {2x_{1} - 3x_{2} } \right)^{2} \times \left( {18 - 32x_{1} + 12x_{1}^{2} + 48x_{2} - 36x_{1} x_{2} + 27x_{2}^{2} } \right)} \right] \hfill \\ \end{gathered}$$	[−5,5]	2	3
$F_{19} = - \sum\limits_{i = 1}^{4} {c_{1} \exp \left( { - \sum\limits_{j = 6}^{3} {a_{ij} (x_{j} } - p_{ij} )^{2} } \right)}$	[1,3]	3	−3.86
$F_{20} = - \sum\limits_{i = 1}^{4} {c_{1} \exp \left( { - \sum\limits_{j = 6}^{6} {a_{ij} (x_{j} } - p_{ij} )^{2} } \right)}$	[0,1]	6	−3.32
$F_{21} = - \sum\limits_{i = 1}^{5} {\left[ {\left( {X - a_{i} } \right)\left( {X - a_{i} } \right)^{T} + c_{i} } \right]^{ - 1} }$	[0,10]	4	−10.1532
$F_{22} = - \sum\limits_{i = 1}^{7} {\left[ {\left( {X - a_{i} } \right)\left( {X - a_{i} } \right)^{T} + c_{i} } \right]^{ - 1} }$	[0,10]	4	10.4028
$F_{23} = - \sum\limits_{i = 1}^{10} {\left[ {\left( {X - a_{i} } \right)\left( {X - a_{i} } \right)^{T} + c_{i} } \right]^{ - 1} }$	[0,10]	4	10.5363

#### Numerical examples with Dim ≤ 30

Each benchmark function runs 30 times by the POA algorithm. A sample size of POA with 30 planets is selected to perform 500 iterations. The statistical results (average–Ave, and standard deviation–Std) are summarized in Tables 2, 3 and 4.

**Table 2:** Results of unimodal benchmark functions.

Fi	POA		PSO		GSA		GWO		ASO		FA		HHO		HGS	
	Aver	Std	Aver	Std	Aver	Std	Aver	Std	Aver	Std	Aver	Std	Aver	Std	Aver	Std
F1	**5.27E−263**	0.00E + 00	1.36E**−**04	2.02E**−**04	2.53E**−**16	9.67E**−**17	6.59E**−**28	6.34E**−**05	2.68E**−**21	3.65E**−**21	1.11E**−**02	3.49E**−**03	3.95E**−**97	1.72E**−**96	2.43E**−**146	1.3E**−**145
F2	**1.08E−137**	4.46E**−**137	4.21E**−**02	4.54E**−**02	5.57E**−**02	1.94E**−**01	7.18E**−**17	2.90E**−**02	3.33E**−**10	1.89E**−**10	2.74E + 01	3.35E + 01	1.56E**−**51	6.98E**−**51	8.16E**−**83	3.80E**−**82
F3	**2.73E−212**	0.00E + 00	7.01E + 01	2.21E + 01	8.97E + 02	3.19E + 02	3.29E**−**06	7.91E + 01	1.98E + 02	7.97E + 01	2.61E + 03	9.84E + 02	1.92E**−**63	1.05E**−**62	5.29E**−**62	2.90E**−**61
F4	**2.45E−124**	1.18E**−**123	1.09E + 00	3.17E**−**01	7.35E + 00	1.74E + 00	5.61E**−**07	1.32E + 00	3.24E**−**09	6.14E**−**09	8.44E**−**02	1.58E**−**02	1.02E**−**47	5.01E**−**47	1.01E**−**66	4.32E**−**66
F5	2.88E + 01	1.48E**−**01	9.67E + 01	6.01E + 01	6.75E + 01	6.22E + 01	2.68E + 01	6.99E + 01	2.48E + 01	5.16E**−**01	7.29E + 04	1.78E + 05	**1.32E−02**	1.87E**−**02	1.44E + 01	1.28E + 01
F6	1.71E**−**01	1.88E**−**01	1.02E**−**04	8.28E**−**05	2.50E**−**16	1.74E**−**16	8.17E**−**01	1.26E**−**04	**0.00E + 00**	0.00E + 00	1.19E**−**02	3.66E**−**03	1.15E**−**04	1.56E**−**04	5.64E**−**06	9.99E**−**06
F7	**1.23E−04**	1.34E**−**04	1.23E**−**01	4.50E**−**02	8.94E**−**02	4.34E**−**02	2.21E**−**03	1.00E**−**01	3.56E**−**02	1.95E**−**02	4.87E**−**02	3.52E**−**02	1.40E**−**04	1.07E**−**04	1.17E**−**03	2.24E**−**03

Significant values are in bold.

**Table 3:** Results of multimodal benchmark functions.

Fi	POA		PSO		GSA		GWO		ASO		FA		HHO		HGS	
	Aver	Std	Aver	Std	Aver	Std	Aver	Std	Aver	Std	Aver	Std	Aver	Std	Aver	Std
F8	−8.64E + 03	6.10E + 02	−4.84E + 03	1.15E + 03	−2.82E + 03	4.93E + 02	−6.12E + 03	−4.09E + 03	−7.43E + 03	4.22E + 02	−6.32E + 03	6.83E + 02	−1.25E + 04	1.47E + 02	−**1.26E + 04**	1.09E + 00
F9	1.44E + 00	7.88E + 00	4.67E + 01	1.16E + 01	2.60E + 01	7.47E + 00	3.11E−01	4.74E + 01	**0.00E + 00**	0.00E + 00	3.24E + 01	9.14E + 00	**0.00E + 00**	0.00E + 00	**0.00E + 00**	0.00E + 00
F10	**8.88E**−**16**	0.00E + 00	2.76E−01	5.09E−01	6.21E−02	2.36E−01	1.06E−13	7.78E−02	3.00E−11	2.15E−11	5.02E−02	1.83E−02	**8.88E**−**16**	4.01E−31	**8.88E**−**16**	0.00E + 00
F11	**0.00E + 00**	0.00E + 00	9.22E−03	7.72E−03	2.77E + 01	5.04E + 00	4.49E−03	6.66E−03	**0.00E + 00**	0.00E + 00	6.05E−03	1.77E−03	**0.00E + 00**	0.00E + 00	**0.00E + 00**	0.00E + 00
F12	2.01E−03	3.50E−03	6.92E−03	2.63E−02	1.80E + 00	9.51E−01	5.34E−02	2.07E−02	**4.51E**−**23**	1.88E−23	2.39E−04	1.18E−04	2.08E−06	1.19E−05	2.10E−07	2.70E−07
F13	1.63E + 00	7.89E−01	6.68E−03	8.91E−03	8.90E + 00	7.13E + 00	6.54E−01	4.47E−03	**1.91E**−**23**	3.12E−22	2.86E−03	1.47E−03	1.57E−04	2.15E−04	6.95E−03	3.80E−02

Significant values are in bold.

**Table 4:** Results of fixed-dimension multimodal benchmark functions.

Fi	POA		PSO		GSA		GWO		ASO		FA			HHO			HGS	
	Aver	Std	Aver	Std	Aver	Std	Aver	Std	Aver	Std	Aver	Std	Aver		Std	Aver		Std
F14	4.76E + 00	3.70E + 00	3.63E + 00	2.56E + 00	5.86E + 00	3.83E + 00	4.04E + 00	4.25E + 00	9.98E−01	7.40E−17	1.88E + 00	6.90E−01	**9.98E**−**01**		9.23E−01	1.32E + 00		1.78E + 00
F15	5.06E−03	1.19E−02	5.77E−04	2.22E−04	3.67E−03	1.65E−03	3.37E−04	6.25E−04	9.47E−04	2.27E−04	1.80E−03	3.54E−03	**3.10E**−**04**		1.97E−04	7.33E−04		1.94E−04
F16	−**1.03E + 00**	4.98E−08	−**1.03E + 00**	6.25E−16	−**1.03E + 00**	4.88E−16	−**1.03E + 00**	−1.03E + 00	−**1.03E + 00**	0.00E + 00	−1.03E + 00	3.19E−09	−1.03E + 00		6.78E−16	−1.03E + 00		6.58E−16
F17	**3.98E**−**01**	7.73E−08	**3.98E**−**01**	0.00E + 00	**3.98E**−**01**	0.00E + 00	3.98E−01	3.98E−01	**3.98E**−**01**	0.00E + 00	3.98E−01	1.01E−09	3.98E−01		2.54E−06	3.98E−01		0.00E + 00
F18	3.90E + 00	4.93E + 00	**3.00E + 00**	1.33E−15	**3.00E + 00**	4.17E−15	3.00E + 00	3.00E + 00	**3.00E + 00**	1.51E−15	3.00E + 00	1.39E−07	**3.00E + 00**		0.00E + 00	**5.70E + 00**		1.48E + 01
F19	−3.81E + 00	1.96E−01	−3.86E + 00	2.58E−15	−3.86E + 00	2.29E−15	−3.86E + 00	−3.86E + 00	−3.86E + 00	2.68E−15	−3.86E + 00	7.74E−10	−3.86E + 00		2.44E−03	−**3.86E + 00**		2.71E−15
F20	−3.25E + 00	1.01E−01	−3.27E + 00	6.05E−02	−3.32E + 00	2.31E−02	−3.29E + 00	−3.25E + 00	−3.32E + 00	1.12E−15	−3.27E + 00	6.92E−02	−**3.32E + 00**		1.37E−01	−3.27E + 00		6.71E−02
F21	−7.37E + 00	3.13E + 00	−6.87E + 00	3.02E + 00	−5.96E + 00	3.74E + 00	−**1.02E + 01**	−9.14E + 00	−8.77E + 00	2.19E + 00	−8.41E + 00	3.21E + 00	−1.01E + 01		8.86E−01	−9.81E + 00		1.29E + 00
F22	−6.35E + 00	3.43E + 00	−8.46E + 00	3.09E + 00	−9.68E + 00	2.01E + 00	−1.04E + 01	−8.58E + 00	−**1.04E + 01**	1.84E−15	−1.01E + 01	1.40E + 00	−1.04E + 01		1.35E + 00	−9.69E + 00		1.84E + 00
F23	−6.38E + 00	3.35E + 00	−9.95E + 00	1.78E + 00	−1.05E + 01	2.60E−15	−1.05E + 01	−8.56E + 00	−1.05E + 01	1.54E−15	−**1.05E + 01**	1.48E−06	−1.05E + 01		9.28E−01	−1.02E + 01		1.37E + 00

Significant values are in bold.

The results from the comparison to F5 and F6 are quite good for POA compared with the others. The F1 to F4 and F7 functions witness that POA algorithm's accuracy is the most superior compared with all other algorithms.

In comparison with 7-unimodal functions, the most of multimodal functions consist of a lots of local optimization areas with the number increasing exponentially with dimensions. This makes them good conditions to evaluate the exploratory ability of a meta-heuristic optimization algorithm.

Table 3 indicates that POA outperforms in F10 and F11 functions, and is quite competitive with the rest.

Similar to the unimodal functions, once again the multimodal and fixed-dimension multimodal functions prove the competitiveness of POA with other algorithms, and show that the obtained results from F14 to F23 are promising.

**Figure 6:**
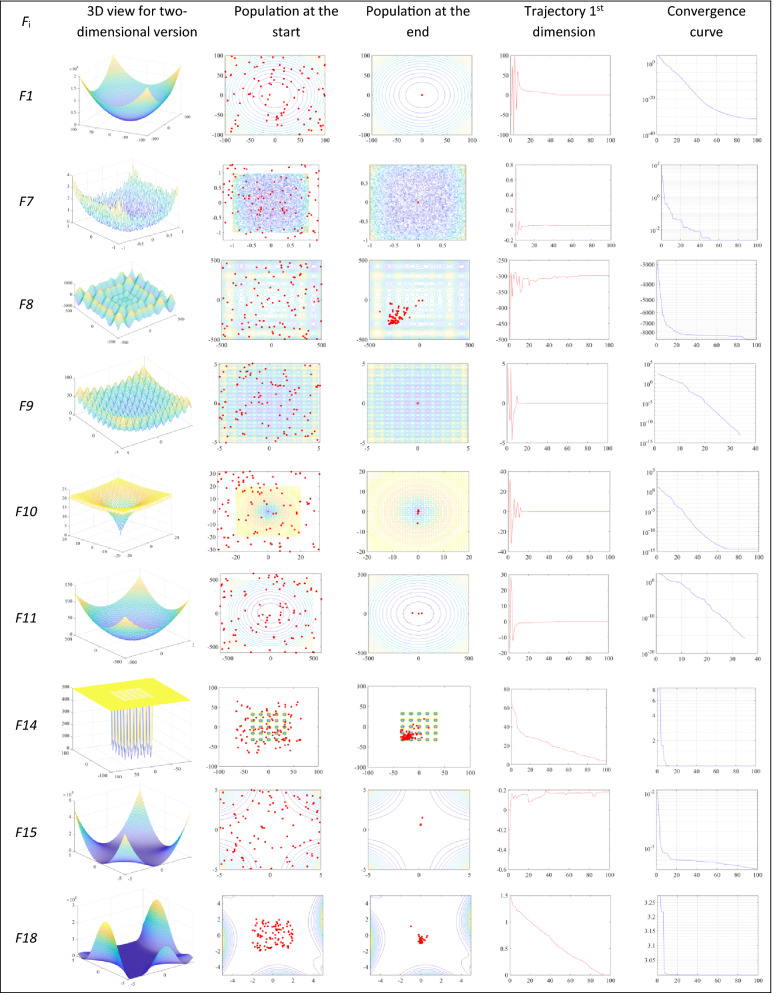
Level of convergence of POA after 100 iterations.

Figure 6 illustrates the convergence of POA after 100 iterations of 100 planets. The first two metrics are qualitative metrics that illustrate the history of planets through the course of generations. During the whole optimization process, the planets are represented using red points as shown in Fig. 6. The trend of planets explores potential zones of the search space, and exploit quite accurately the global optimum. These investigations demonstrate that POA is able to get the high effectiveness in approximating the global optimum of optimization problems.

The third metric presents the movement of the 1st planet in the first dimension during optimization. This metric helps us to monitor if the first planet, which represents all planets, faces sudden movement in the initial generations and has more stability in the final generations. This movement is able to guarantee the exploration of the search region. Finally, the movement of planets is very short, which causes exploitation of the search region. Obviously, POA demonstrates that this is an algorithm that meets a requirement of accuracy, as well as a high degree of convergence.

The final quantitative metric is the convergence level of the POA algorithm. The best value of all planets in each generation is stored and the convergence curves are shown in Fig. 6. The decreasing of fitness over the generations demonstrates the convergence of the POA algorithm.

#### Numerical examples with high-dimensional optimization problems

To validate the performance of POA with respect to high-dimensional optimization problems, the first 13 classical benchmark functions of the above-mentioned ones with Dim = 1000 are employed to investigate POA. For a fair comparison, seven of the above mentioned meta-heuristic optimization algorithms and POA with population size *N* = 30 independently run in 30 times. Additionally, the maximum number of iterations is fixed at 500 for all test functions.

**Figure 7:**
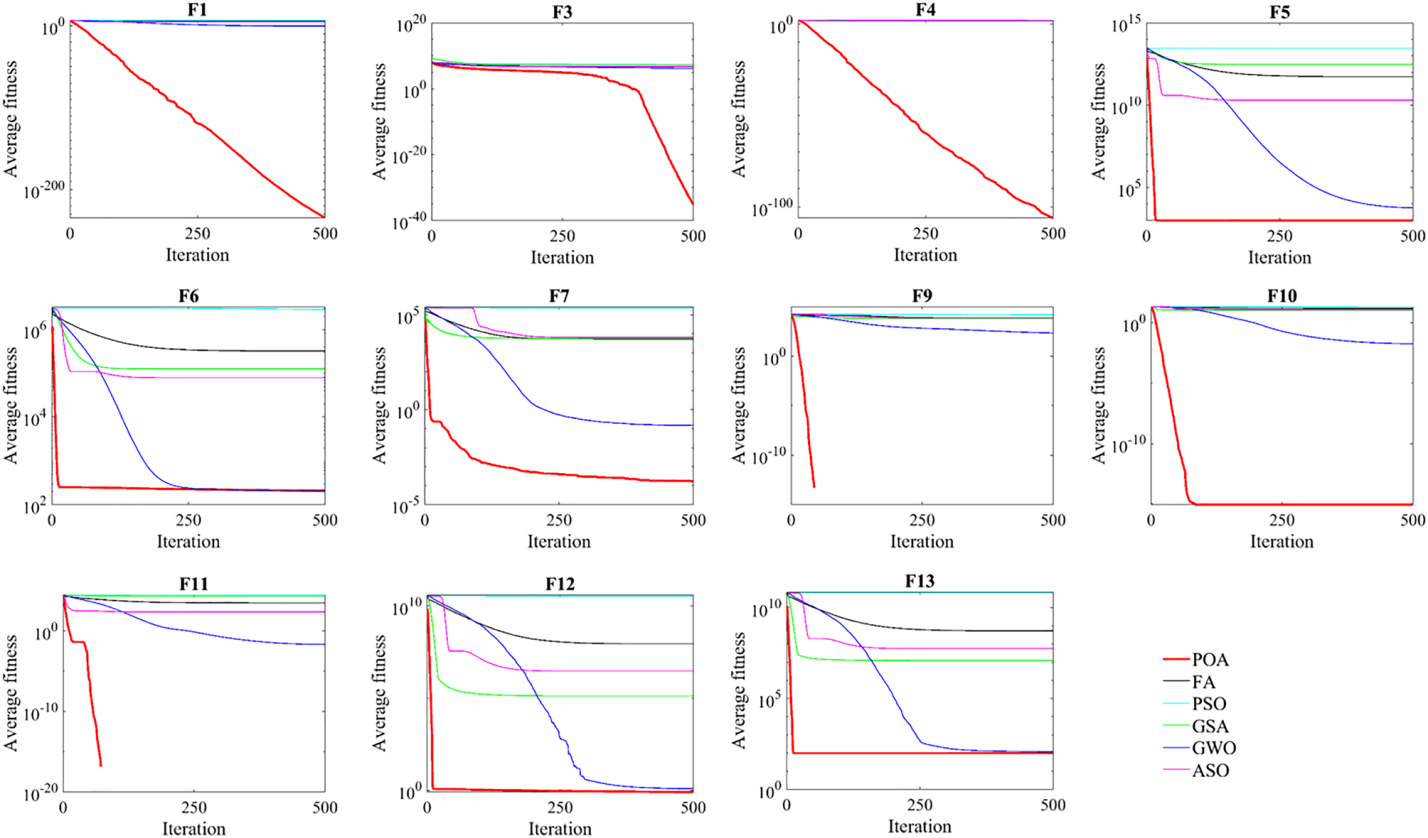
Performance comparison of algorithms.

The test discussed in this subsection demonstrate that POA is promising for dealing with 13 classical benchmark problems. Among the tested 23 benchmark functions, 13 functions had Dim = 1000, as presented in Table 5 and Fig. 7. This subsection confirmed the ability of POA to deal with high-dimensional problems as the dimension of those 13 classical benchmarks has been increased from 30 to 1000.

**Table 5:** Results of the first 13 benchmark functions with Dim = 1000.

Fi	POA		PSO		GSA		GWO		ASO		FA		HHO		HGS	
	Aver	Std	Aver	Std	Aver	Std	Aver	Std	Aver	Std	Aver	Std	Aver	Std	Aver	Std
F1	**1.84E**−**235**	0	2,907,289	144,955	126,322.75	3842.509	0.234087	0.066678	82,745.994	12,030.5	329,872.3	27,485.71	6.46E−94	3.38E−93	6E−129	3.5E−128
F2	**9.66E**−**130**	5.3E−129	2.4E + 109	1.3E + 110	806.94152	64.64325	4.94E−07	1.91E−07	1434.0685	170.13	790.4466	177.5697	6.36E−49	3.27E−48	65,535	−
F3	**7.191E**−**36**	3.94E−35	25,598,720	3,619,734	26,256,697	15,611,478	1,587,902	256,574.3	5,792,444.1	1,480,296	6,497,218	981,568.6	4.12E−16	2.26E−15	1,302,171	3,079,055
F4	**1.07E**−**106**	4.5E−106	99.60416	0.126364	35.202087	1.60048	79.1974	3.345111	66.011183	7.04421	95.07072	0.824173	3.34E−47	1.32E−46	6.2E−58	3.41E−57
F5	998.90393	0.060544	2.89E + 13	5.65E + 11	3.102E + 12	5.17E + 10	5989.334	1851.481	2.013E + 10	6.9E + 09	5.48E + 11	6.58E + 10	**3.76E**−**01**	6.99E−01	462.198	501.1158
F6	207.83991	6.874046	2,867,223	179,732.3	125,969.19	5541.92	203.1294	2.189645	78,694.683	10,546.7	324,249.3	21,365.01	5.95E−03	6.43E−03	**0.00427**	0.008167
F7	0.000175	0.000123	240,456.4	5595.043	5621.5092	684.4318	0.146884	0.02925	6438.0108	740.576	5186.77	623.5121	**1.67E**−**04**	1.65E−04	0.00073	0.001021
F8	−106,369.8	7637.524	−96,426.4	4616.017	−130.8805	882.5566	−85,658.3	18,724.48	−38,868.24	4456.87	−126,173	9306.83	−**4.19E + 05**	4.15E + 01	−406,482	36,013.5
F9	**0**	0	15,435.68	765.3185	6588.5853	198.471	221.053	60.17693	7518.9635	207.096	7384.177	220.0783	**0**	0	**0**	0
F10	**8.882E**−**16**	0	20.70236	0.423177	10.911026	0.180167	0.018329	0.002733	12.466618	0.24204	15.5555	0.19581	**8.88E**−**16**	0.00E + 00	**8.88E**−**16**	0
F11	**0**	0	26,569.83	1540.641	21,603.807	136.2224	0.020375	0.028575	214.13913	15.3359	2928.102	214.1877	**0**	0	**0**	0
F12	0.7970368	0.036669	3.63E + 10	1.02E + 09	133,193.57	108,367.3	1.232015	0.285816	2,928,355.2	2,134,590	88,534,017	18,592,961	**1.47E**−**06**	2.11E−06	0.03011	0.16487
F13	99.567897	0.180338	6.62E + 10	1.59E + 09	12,570,826	2,297,967	119.6103	6.061993	55,894,267	2.2E + 07	5.36E + 08	95,379,577	**1.30E**−**03**	1.43E−03	9.83355	29.99756

Significant values are in bold.

#### Wall-clock time analysis

In this experiment, a comparison is made between POA and the other seven algorithms in the time-consuming computation experiments of the 13 functions. The time-consuming calculation method is that each benchmark function independently implements 30-times all algorithms, then the values of 30-time running is saved in Table 6. For Dim = 30, not only does the computation of POA outperform some algorithms, while taking less time, such as GSA, ASO, and FA, but also it is sometimes far superior to GWO, even the time-consuming calculation of PSO. For Dim = 1000, POA always ranks first in computational time. These results show that the POA has merit for optimization problems in high dimensional problems.

**Table 6:** Wall-clock time costs (second) on benchmarks of POA and other participants.

Fi	Dim = 30								Dim = 1000							
	FA	GSA	ASO	PSO	GWO	HHO	HGS	POA	FA	GSA	ASO	PSO	GWO	HHO	HGS	POA
F1	6.52	7.51	11.45	1.47	2.71	1.69	1.78	1.74	72.65	150.28	91.24	11.16	51.21	10.77	35.28	8.21
F2	6.31	7.56	11.46	1.52	2.84	1.56	1.88	1.79	52.92	1703.72	3853.03	11.56	51.76	11.36	32.64	8.62
F3	8.44	9.59	13.65	3.79	5.09	8.40	4.04	4.03	785.54	933.45	756.52	326.68	364.90	755.73	344.42	326.83
F4	6.07	7.31	11.10	1.41	2.69	1.95	1.69	1.68	126.96	311.10	196.63	15.25	105.74	12.76	30.90	8.23
F5	6.38	7.59	11.45	1.71	2.97	3.09	2.01	1.94	104.48	328.69	201.28	22.47	108.81	18.67	32.05	12.88
F6	6.08	7.31	11.16	1.41	2.68	2.30	1.70	1.67	154.86	336.07	209.36	21.30	95.95	15.99	31.44	8.38
F7	7.64	8.73	12.76	2.89	4.17	5.36	3.19	3.14	189.12	333.90	223.45	55.09	76.02	52.57	49.73	39.10
F8	6.45	7.60	11.45	1.76	3.09	4.04	2.03	2.07	170.21	333.92	208.38	36.22	114.84	32.51	37.06	26.53
F9	6.35	7.47	11.95	1.67	2.82	2.69	3.60	1.69	78.21	154.78	95.58	17.93	53.37	24.50	362.97	12.00
F10	6.37	7.47	11.45	1.73	2.81	2.81	3.21	1.76	78.86	158.87	95.81	17.60	52.84	25.08	361.28	13.66
F11	7.21	8.25	14.26	1.96	3.04	3.25	3.74	1.99	102.20	157.92	109.01	20.61	55.02	30.96	417.91	14.60
F12	10.21	11.25	15.33	5.55	6.70	12.24	5.75	5.82	114.12	188.42	131.18	53.23	90.17	113.10	152.32	48.53
F13	10.35	11.33	15.38	5.60	6.70	12.28	5.75	5.79	114.48	189.23	131.45	53.13	89.94	115.27	171.67	43.90
Sum	94.39	108.95	162.88	32.47	48.30	61.65	40.37	35.11	2144.60	5280.36	6302.91	662.26	1310.59	1219.29	2059.67	571.47
Ranking	6	7	8	1	4	5	3	2	6	7	8	2	4	3	5	1

### Experimental results using CEC functions

In order to further clarify the efficiency of the proposed algorithm, POA is tested on the complex challenges, namely Evaluation Criteria for the CEC 2017^30^ and CEC 2019^31^. Its results are compared with those of well-known and modern meta-heuristic algorithms: DA, WOA, and the arithmetic optimization algorithm (AOA)^32^. These algorithms are selected because of the reasons:

- All of them are based on the principle of PSO as with POA.
- All algorithms are well cited in the literature, and AOA is a recently published study.
- These algorithms were proven that they were superior performance both on benchmark test functions and real-world problems.
- They are publicly provided by their authors.

Like the 23 classical benchmark functions, each function of the CEC Benchmark Suite is run 30 times, and each algorithm was allowed to search the landscape for 500 iterations using 30 agents.

#### CEC 2017 problems

In this subsection, the IEEE CEC 2017 problems is employed to test the performance of POA. The CEC'17 standard set consists of 28 real challenging benchmark problems. The first is unimodal function, 2–7 are multimodal one. While ten functions next are Hybrid, the rest of CEC 2017 are 10 composition functions. Table 7 presents a brief description of CEC 2017.

**Table 7:** CEC 2017 problems.

Type function	Fi	Function name	Range	Dim	Min_*F*_
Unimodal functions	F24	Shifted and Rotated Bent Cigar Function	[−100,100]	10	100
Simple multimodal functions	F25	Shifted and Rotated Rosenbrock’s Function			300
	F26	Shifted and Rotated Rastrigin’s Function			400
	F27	Shifted and Rotated Expanded Scaffer’s F7 Function			500
	F28	Shifted and Rotated Lunacek Bi_Rastrigin Function			600
	F29	Shifted and Rotated Non-Continuous Rastrigin’s Function			700
	F30	Shifted and Rotated Levy Function			800
	F31	Shifted and Rotated Schwefel’s Function			900
Hybrid functions	F32	Hybrid Function 1 (*N* = 3)			1000
	F33	Hybrid Function 2 (*N* = 3)			1100
	F34	Hybrid Function 3 (*N* = 3)			1200
	F35	Hybrid Function 4 (*N* = 4)			1300
	F36	Hybrid Function 5 (*N* = 4)			1400
	F37	Hybrid Function 6 (*N* = 4)			1500
	F38	Hybrid Function 7 (*N* = 5)			1600
	F39	Hybrid Function 8 (*N* = 5)			1700
	F40	Hybrid Function 9 (*N* = 5)			1800
	F41	Hybrid Function 10 (*N* = 6)			1900
Composition functions	F42	Composition Function 1 (*N* = 3)			2000
	F43	Composition Function 2 (*N* = 3)			2100
	F44	Composition Function 3 (*N* = 4)			2200
	F45	Composition Function 4 (*N* = 4)			2300
	F46	Composition Function 5 (*N* = 5)			2400
	F47	Composition Function 6 (*N* = 5)			2500
	F48	Composition Function 7 (*N* = 6)			2600
	F49	Composition Function 8 (*N* = 6)			2700
	F50	Composition Function 9 (*N* = 3)			2800
	F51	Composition Function 10 (*N* = 3)			2900

As shown in Table 8, POA is highly efficient, because compared to WOA, DA and AOA, it outperforms all algorithms in 21/28 of CEC 2017 standard set. In addition, the Wilcoxon signed rank test with *α* = 0.05 significance level is shown in Table 9 in order to analyze the significant differences between the results of POA and other algorithms. These results have proven that POA provides a great performance in terms of solution quality when handling the functions of CEC 2017.

**Table 8:** Results of CEC 2017 problems.

Fi	Measured	POA	WOA	DA	AOA	Fi	Measure	POA	WOA	DA	AOA
F24	Worst	**21,616.689**	446,086,810.967	1,614,920,665.975	17,989,949,690.271	F38	Worst	**2207.641**	2293.244	2342.246	2420.476
	Best	**2369.991**	2,437,147.665	95,752.700	3,713,067,906.824		Best	**1601.839**	1671.161	1725.183	1635.551
	Aver	**9077.871**	78,794,903.555	121,819,809.036	9,746,903,748.119		Aver	**1914.943**	1927.995	1966.959	2031.060
	Std	**4926.262**	105,264,259.045	295,134,280.931	3,636,949,495.724		Std	162.953	**149.276**	154.805	165.504
F25	Worst	**329.886**	14,038.326	42,054.108	18,439.573	F39	Worst	2034.458	**1981.868**	1984.712	2134.607
	Best	**300.020**	1212.600	820.018	9737.963		Best	**1724.880**	1756.245	1757.774	1761.719
	Aver	**303.572**	6419.583	8055.177	14,013.305		Aver	**1789.454**	1820.757	1836.497	1878.853
	Std	**8.346**	4312.887	9311.269	2659.791		Std	63.748	62.726	**61.045**	104.133
F26	Worst	**484.521**	605.709	570.901	2474.368	F40	Worst	55,482.667	**36,953.291**	55,541.892	209,928,837.936
	Best	**400.004**	405.435	403.243	479.108		Best	2917.407	**2181.715**	2364.981	2361.926
	Aver	**411.796**	442.972	448.726	1171.863		Aver	20,533.715	**17,197.131**	20,863.963	7,307,877.076
	Std	**20.353**	47.700	44.320	562.736		Std	15,384.264	**11,293.771**	16,797.329	38,300,874.924
F27	Worst	**594.526**	599.904	633.492	606.939	F41	Worst	**22,421.717**	2,451,752.098	89,900.455	237,845.333
	Best	522.888	**509.439**	529.121	529.864		Best	2058.212	2099.614	**1968.492**	4665.629
	Aver	**547.603**	554.825	563.587	563.214		Aver	**9793.255**	126,429.570	20,811.902	104,654.601
	Std	**16.035**	21.518	23.620	20.047		Std	**6755.212**	448,601.416	25,868.597	78,412.954
F28	Worst	661.566	672.090	680.871	**658.735**	F42	Worst	2399.638	2358.672	2344.633	**2290.265**
	Best	**604.546**	606.142	609.318	621.384		Best	**2052.699**	2063.242	2065.773	2062.513
	Aver	**626.008**	640.832	635.708	639.634		Aver	2194.909	2175.757	2194.011	**2160.552**
	Std	14.833	17.992	14.974	**8.022**		Std	91.767	83.828	78.431	**69.544**
F29	Worst	821.235	835.056	**771.432**	826.021	F43	Worst	2391.776	2422.329	2392.790	**2389.428**
	Best	730.816	739.591	**714.955**	766.210		Best	**2200.022**	2207.964	2205.519	2232.131
	Aver	768.674	785.295	**745.035**	801.622		Aver	2330.843	2323.952	**2321.882**	2328.732
	Std	20.930	27.487	14.838	**14.756**		Std	54.209	63.445	64.072	**43.707**
F30	Worst	863.684	877.893	872.106	**859.711**	F44	Worst	3780.977	4001.830	**2377.450**	3667.396
	Best	814.926	**812.180**	813.115	820.715		Best	**2242.956**	2250.448	2252.663	2390.081
	Aver	**836.686**	845.449	838.638	841.861		Aver	2428.039	2527.315	**2331.853**	2988.301
	Std	12.725	19.555	14.283	**9.177**		Std	383.046	496.162	**25.432**	298.582
F31	Worst	1775.443	2755.601	2974.621	**1761.634**	F45	Worst	**2701.334**	2711.230	2740.647	2850.536
	Best	941.832	977.617	**920.630**	1016.576		Best	2626.399	2623.470	**2623.180**	2688.632
	Aver	**1233.102**	1582.821	1329.104	1445.874		Aver	**2647.105**	2662.015	2686.121	2752.342
	Std	229.408	356.906	458.930	**222.809**		Std	**18.782**	22.188	29.912	39.502
F32	Worst	**2805.144**	2874.741	3177.011	2845.738	F46	Worst	2853.043	**2842.332**	2882.747	3026.458
	Best	**1460.956**	1595.143	1462.482	1843.645		Best	**2500.121**	2614.982	2756.338	2658.237
	Aver	**2124.758**	2178.843	2300.517	2267.726		Aver	**2760.174**	2782.705	2814.403	2854.468
	Std	329.156	346.841	396.083	**248.939**		Std	75.666	39.952	**33.775**	78.993
F33	Worst	**1394.636**	1456.809	1553.285	11,284.167	F47	Worst	**3024.368**	3057.139	3281.954	4031.440
	Best	**1109.370**	1127.720	1137.880	1170.419		Best	**2897.766**	2926.524	2901.047	3091.840
	Aver	**1177.684**	1264.158	1305.746	4259.407		Aver	**2938.987**	2970.737	2974.748	3433.472
	Std	**64.587**	97.652	105.699	2742.167		Std	32.948	**30.706**	73.376	240.484
F34	Worst	**2,640,627.083**	23,239,770.178	23,841,149.599	1,410,137,474.156	F48	Worst	**4405.324**	4581.077	4530.516	4693.227
	Best	**9420.672**	75,356.443	12,974.629	37,495.771		Best	**2802.188**	3108.297	2805.272	3395.848
	Aver	**426,091.817**	7,244,799.337	5,290,868.507	210,043,229.615		Aver	3542.206	3657.193	**3371.774**	4098.304
	Std	**541,693.197**	6,903,050.501	5,851,002.051	299,766,603.285		Std	556.018	513.032	528.992	**291.224**
F35	Worst	**32,516.647**	88,773.203	83,425.506	34,873.960	F49	Worst	**3205.502**	3238.281	3282.543	3407.872
	Best	2033.414	1701.946	**1639.672**	3600.132		Best	**3089.526**	3097.429	3097.862	3165.500
	Aver	**6943.151**	22,033.157	21,794.756	11,494.178		Aver	**3135.637**	3147.792	3144.096	3261.204
	Std	**8029.349**	21,046.434	19,381.270	8648.765		Std	**38.055**	44.878	50.332	65.214
F36	Worst	27,866.569	**7441.839**	10,005.432	27,668.496	F50	Worst	3749.371	3736.181	**3731.813**	3954.137
	Best	**1434.406**	1493.885	1512.187	1468.538		Best	**3100.397**	3150.013	3171.896	3481.000
	Aver	10,725.802	2937.645	**2890.960**	11,460.443		Aver	**3362.144**	3444.478	3364.905	3783.707
	Std	9776.325	**1746.346**	2099.952	9589.028		Std	148.755	179.170	**118.383**	120.887
F37	Worst	**6053.120**	33,587.791	98,852.122	34,674.857	F51	Worst	3588.233	3706.539	**3520.137**	3764.012
	Best	**1572.781**	2339.039	3132.247	4775.219		Best	**3146.758**	3193.109	3149.620	3184.949
	Aver	**3710.869**	12,292.528	26,066.915	18,618.951		Aver	3318.534	3377.156	**3300.440**	3435.494
	Std	**1458.299**	8834.587	24,462.365	6297.956		Std	112.592	120.658	**80.176**	167.909

Significant values are in bold.

**Table 9:** Results of Wilcoxon sign-rank test for CEC 2017 problems with α = 0.05.

Fi	POA vs WOA		POA vs DA		POA vs AOA	
	*p*-value	Winner	*p*-value	Winner	*p*-value	Winner
F24	3.02E−11	+	3.02E−11	+	3.02E−11	+
F25	3.02E−11	+	3.02E−11	+	3.02E−11	+
F26	2.20E−07	+	1.73E−07	+	3.34E−11	+
F27	2.34E−01	~	3.50E−03	+	3.50E−03	+
F28	1.37E−03	+	1.99E−02	+	2.13E−04	+
F29	2.71E−02	+	2.00E−05	−	8.35E−08	+
F30	1.12E−01	~	7.73E−01	~	4.84E−02	+
F31	3.83E−05	+	9.94E−01	~	6.55E−04	+
F32	5.30E−01	~	7.98E−02	~	7.98E−02	~
F33	1.32E−04	+	2.49E−06	+	2.15E−10	+
F34	1.61E−06	+	1.25E−05	+	6.12E−10	+
F35	4.22E−04	+	1.32E−04	+	1.41E−04	+
F36	1.70E−02	−	1.56E−02	−	5.79E−01	~
F37	7.04E−07	+	5.00E−09	+	2.15E−10	+
F38	8.42E−01	~	2.58E−01	~	1.17E−02	+
F39	1.08E−02	+	4.22E−04	+	3.16E−05	+
F40	3.79E−01	~	7.96E−01	~	7.06E−01	~
F41	5.83E−03	+	3.48E−01	~	8.35E−08	+
F42	4.38E−01	~	8.77E−01	~	1.91E−01	~
F43	7.73E−01	~	8.77E−01	~	5.01E−01	~
F44	1.78E−04	+	4.64E−05	–	6.53E−08	+
F45	5.83E−03	+	8.20E−07	+	4.50E−11	+
F46	9.33E−02	~	4.08E−05	+	9.51E−06	+
F47	1.53E−05	+	1.30E−03	+	3.02E−11	+
F48	5.37E−02	~	2.52E−01	~	3.37E−04	+
F49	2.12E−01	~	4.12E−01	~	1.41E−09	+
F50	1.17E−02	+	2.84E−01	~	1.78E−10	+
F51	5.55E−02	~	8.53E−01	~	6.10E−03	+
Sum (+ / ~ /−)	16/11/1		13/12/3		23/5/0	

#### CEC 2019 problems

Table 10 presents a brief description of CEC 2019. It can be seen from Table 11 that POA outperforms other optimization algorithms in all CEC 2019 functions. Indeed, results in many test functions (e.g. F52, F53, F56) show that POA is more powerful than others not only at the average value of 30 runs, but also at the other statistical values, such as the best, worst and Std value. Once again, The Wilcoxon signed rank test (as shown in Table 12) demonstrated the superior performance of POA to solve CEC 2019 problems.

**Table 10:** CEC 2019 problems.

Fi	Function name	Range	Dim	Min_F_
F52	Storn's Chebyshev Polynomial Fitting Problem	[−8192, 8192]	9	1
F53	Inverse Hilbert Matrix Problem	[−16384, 16384]	16	1
F54	Lennard–Jones Minimum Energy Cluster	[−4,4]	18	1
F55	Rastrigin’s Function	[−100,100]	10	1
F56	Griewangk’s Function	[−100,100]	10	1
F57	Weierstrass Function	[−100,100]	10	1
F58	Modified Schwefel’s Function	[−100,100]	10	1
F59	Expanded Schaffer’s F6 Function	[−100,100]	10	1
F60	Happy Cat Function	[−100,100]	10	1
F61	Ackley Function	[−100,100]	10	1

**Table 11:** Results of CEC 2019 problems.

Fi	Measure	POA	WOA	DA	AOA	Fi	Measure	POA	WOA	DA	AOA
F52	Worst	**1383.962**	1.22E + 08	87,793,661	47,045,606	F57	Worst	**10.35371**	11.98996	11.97721	13.24428
	Best	**1**	3833.742	43,190.53	1		Best	**2.578827**	6.74338	3.858144	7.601071
	Aver	**49.64264**	16,848,021	22,172,756	1,783,859		Aver	**6.845989**	9.124547	7.868689	10.27923
	Std	**252.1988**	23,542,448	19,745,213	8,567,816		Std	1.882445	**1.349568**	1.754316	1.448363
F53	Worst	**1005.32**	10,766.06	10,927.26	18,365.92	F58	Worst	**1728.034**	1827.559	2059.978	1786.957
	Best	**4.567066**	1311.151	1092.686	3216.332		Best	614.7954	**391.1025**	932.6491	957.0947
	Aver	**143.2106**	6503.912	6160.632	10,895.68		Aver	**1256.916**	1265.31	1475.388	1403.671
	Std	**231.2333**	2678.268	2673.48	3519.805		Std	326.6572	337.8082	329.3905	**199.6095**
F54	Worst	10.71197	**9.708243**	11.7112	11.6667	F59	Worst	5.083027	5.240865	**5.079878**	5.300394
	Best	**1.410337**	2.832679	5.731269	8.944851		Best	3.739129	3.956049	**3.703836**	4.082092
	Aver	**5.985024**	6.151234	10.2168	10.44986		Aver	**4.577152**	4.67067	4.679051	4.765198
	Std	2.954948	1.936269	1.307939	**0.795213**		Std	0.360475	0.365394	**0.298852**	0.329205
F55	Worst	**79.6702**	96.71619	101.0834	101.8834	F60	Worst	**1.743374**	1.822469	1.85515	3.90721
	Best	18.91121	22.50731	**16.74522**	26.98737		Best	1.144939	1.16266	**1.119194**	1.562143
	Aver	**41.42796**	54.74208	55.51705	58.95477		Aver	**1.36822**	1.414833	1.427379	3.081278
	Std	**15.33676**	22.97717	21.37947	19.73287		Std	**0.145476**	0.174602	0.212619	0.654018
F56	Worst	**2.081627**	5.37188	29.52093	152.7949	F61	Worst	21.40822	21.63267	21.57491	**21.21511**
	Best	**1.093455**	1.943486	1.150722	30.21018		Best	21.00662	21.07732	**20.99995**	21.07513
	Aver	**1.355729**	2.709666	3.326794	85.96353		Aver	**21.08235**	21.27699	21.26729	21.12679
	Std	**0.246973**	0.775102	5.265839	28.65205		Std	0.085093	0.151047	0.145117	**0.033882**

Significant values are in bold.

**Table 12:** Results of Wilcoxon sign-rank test for CEC 2019 problems with α = 0.05.

Fi	F52	F53	F54	F55	F56	F57	F58	F59	F60	F61	Sum (+ / ~ /−)
**POA vs WOA**											
*p*-value	1.62E−11	3.02E−11	5.89E−01	2.61E−02	4.98E−11	7.22E−06	8.77E−01	2.28E−01	3.55E−01	6.01E−08	6/4/0
Winner	+	+	~	+	+	+	~	~	~	+	
**POA vs DA**											
*p*-value	1.62E−11	3.02E−11	3.65E−08	6.38E−03	6.28E−06	4.51E−02	2.92E−02	3.33E−01	4.20E−01	2.68E−06	8/2/0
Winner	+	+	+	+	+	+	+	~	~	+	
**POA vs AOA**											
*p*-value	1.47E−09	3.02E−11	1.41E−09	3.37E−04	3.02E−11	1.56E−08	9.33E−02	3.92E−02	4.50E−11	5.87E–04	9/1/0
Winner	+	+	+	+	+	+	~	+	+	+	

In the next section, some classical engineering design problems are employed to further evaluate the performance of the POA. Besides, POA is also compared with other well-known techniques to confirm its results.

### Engineering design problems

In this study, three constrained engineering design problems, namely tension/compression spring, welded beam, pressure vessel designs, are used to investigate the applicability of POA. The problems have some equality and inequality constraints. The POA should be, therefore, equipped with a constraint solving technique. Meanwhile, POA can optimize constrained problems as well at the same time. It should be noted that the population size and the number of iterations are, respectively, set to 30 and 500 for 50 runs to find the results for all problems in this section.

#### Tension/compression spring

The main aim of this problem is to minimize the weight of a tension/compression spring. The design problem is subject to three constraints, namely surge frequency, shear stress, and minimum deflection. This problem consists of three variables: Wire diameter (*d*), mean coil diameter (*D*), and the number of active coils (*N*).

Tension/compression spring design problem has been solved by both mathematicians and heuristic techniques. Some researchers have made efforts to employ several methods for minimizing the weight of a tension/compression spring (Ha and Wang: PSO^33^; Coello and Montes: The Evolution Strategy (ES)^34^ and GA^35^; Mahdavi et al.: Harmony Search (HS)^36^; Belegundu: Mathematical optimization^37^ and Arora: Constraint correction^38^; Huang et al.: Differential Evolution (DE)^39^). Additionally, GWO^5^ algorithms and HHO^11^ have also been employed as heuristic optimizers for this problem. The comparison of the results of these methods and POA is shown in Table 13.

**Table 13:** Comparison of results for tension/compression spring.

Candidates	Optimum variables			Optimum weight
	*d*	*D*	*N*	
HHO	0.05179639	0.35930536	11.138859	0.01266544
POA	**0.051767**	**0.358602**	**11.179891**	**0.01266588**
GWO	0.051690	0.356737	11.288850	0.01266600
MFO	0.051994	0.364109	10.868422	0.01266690
DE (Huang et al.)	0.051609	0.354714	11.410831	0.01267020
HS (Mahdavi et al.)	0.051154	0.349871	12.076432	0.01267060
PSO (Ha and Wang)	0.051728	0.357644	11.244543	0.01267470
ES (Coello and Montes)	0.051989	0.363965	10.890522	0.01268100
GSA	0.050276	0.323680	13.525410	0.01270220
GA (Coello)	0.051480	0.351661	11.632201	0.01270480
Mathematical optimization (Belegundu)	0.053396	0.399180	9.185400	0.01273030
Constraint correction (Arora)	0.050000	0.315900	14.250000	0.01283340

Significant values are in bold.

#### Welded beam

The aim of welded beam design problem is to minimize its fabrication cost. The constraints of the problem are shear stress $(\tau )$, bending stress in the beam $(\theta )$, buckling load of the bar $(P_{c} )$, end deflection of the beam $(\delta )$ and side constraints. Welded beam design problem has four variables, namely thickness of weld $(h)$, length of attached part of bar $(l)$, the height of the bar $(t)$, and thickness of the bar $(b)$. This problem is illustrated in the literature^5,40,41^.

Lee and Geem^40^ employed HS to deal with this problem, while Deb^42,43^ and Coello^44^ used GA. Seyedali Mirjalili applied GWO^5^ to solve this problem. Richardson’s random approach, Davidon-Fletcher-Powell, Simplex technique, Griffith and Stewart’s successive linear approximation are the mathematical methods that have been adopted by Ragsdell and Philips^41^ for this problem. More recently, Heidari, et al.^11^ and Yang, et al.^29^ have used HHO and HGS, respectively, to solve the problem. Table 14 shows a comparison between the different methods. The results indicate that POA reaches a design with the minimum cost compared to other optimizations. The best result of the cost function obtained by the POA is **1.72564.**

**Table 14:** Comparison of results for welded beam design problem.

Candidates	Optimum variables				Optimum cost
	$(h)$	$(l)$	$(t)$	$(b)$	
**POA**	**0.20563**	**3.47242**	**9.03821**	**0.20578**	**1.72564**
GWO	0.20568	3.47838	9.03681	0.205778	1.726240
HHO	0.204039	3.531061	9.027463	0.206147	1.731991
GA Coello)	N.A	N.A	N.A	N.A	1.824500
GSA	0.182129	3.856979	10	0.202376	1.879952
HGS	0.26	5.1025	8.03961	0.26	2.302076
GA (Deb)	N.A	N.A	N.A	N.A	2.380000
HS (Lee and Geem)	0.2442	6.2231	8.2915	0.2443	2.380700
APPROX	0.2444	6.2189	8.2915	0.2444	2.381500
David	0.2434	6.2552	8.2915	0.2444	2.384100
GA (Deb)	0.2489	6.173	8.1789	0.2533	2.433100
Simplex	0.2792	5.6256	7.7512	0.2796	2.530700
Random	0.4575	4.7313	5.0853	0.6600	4.118500

Significant values are in bold.

#### Pressure vessel

Pressure vessel design problem is well-known, where the fabrication cost of the total cost consisting of material, forming, and welding of a cylindrical vessel should be minimized. There are four variables, namely thickness of the shell $(T_{s} )$, thickness of the head $(T_{h} )$, Inner radius $(R)$ and length of the cylindrical section without considering the head $(L)$, and four constraints.

Pressure vessel design problem has also been popular among optimization studies in different researches. Several heuristic techniques, namely DE^39^, PSO^33^, GA^35,45,46^, ACO^47^, ES [59], GWO^5^, MFO^48^, HHO^11^ and SMA^10^, that have been adopted for the optimization of this problem. Mathematical approaches employed are augmented Lagrangian Multiplier^49^ and branch-and-bound^50^. We can see that POA is again able to search a design with the minimum cost as shown in Table 15.

**Table 15:** Comparison of results for pressure vessel design problem.

Candidates	Optimum variables				Optimum cost
	$(T_{s} )$	$(T_{h} )$	$(R)$	$(L)$	
POA	0.7832	0.3873	40.5769	196.4752	5895.4160
SMA	0.7931	0.3932	40.6711	196.2178	5994.1857
HHO	0.81758383	0.4072927	42.09174576	176.7196352	6000.4626
GWO	0.8125	0.4345	42.089181	176.758731	6051.5639
ACO (Kaveh and Talataheri)	0.8125	0.4375	42.103624	176.572656	6059.0888
MFO	0.8125	0.4375	42.098445	176.636596	6059.7143
DE (Huang et al.)	0.8125	0.4375	42.098411	176.637690	6059.7340
ES (Montes and Coello)	0.8125	0.4375	42.098087	176.640518	6059.7456
GA (Coello and Montes)	0.8125	0.4375	42.097398	176.654050	6059.9463
PSO (He and Wang)	0.8125	0.4375	42.091266	176.746500	6061.0777
GA (Coello)	0.8125	0.4345	40.323900	200.000000	6288.7445
GA (Deb and Gene)	0.9375	0.5000	48.329000	112.679000	6410.3811
Lagrangian Multiplier (Kannan)	1.1250	0.6250	58.291000	43.690000	7198.0428
Branch-bound (Sandgren)	1.1250	0.6250	47.700000	117.701000	8129.1036
GSA	1.1250	0.6250	55.988660	84.454203	8538.8359

## Conclusions

In the paper, a meta-heuristic algorithm, inspired by the gravitational law of Newton, is proposed. POA's structure in search processes consists of 2 phases that aim for proper balance exploration and exploitation. Several outperform features are shown through the accuracy of 23 classical benchmark functions and 38 IEEE CEC test functions (CEC2017, CEC 2019). In many functions, POA showed that the obtained results are more accurate than the others many times.

In the final evaluation section, a set of well-known test cases, including three engineering test problems, are thoroughly investigated to examine the operation of POA in practice. Each problem is a type of distinct engineering, including very diverse search spaces. Therefore, these engineering problems are employed to test the POA thoroughly. The obtained results demonstrate that POA is able to solve effectively real challenging problems with unknown search spaces and a large number of constraints. The results compared to GSA, GWO, PSO, DE, ACO, MFO, SOS, CS, HHO, SMA, HGS, etc., suggest that POA is superior.

The structure of POA is simple and explicit, very effective, even fast. Experiments revealed short computational time for handling complex optimization problems. Therefore, we firmly authenticate that POA is a powerful algorithm to solve optimization problems.

## Data Availability

All data generated or analyzed during this study are included in this published article.

## References

[1] Kennedy, J. & Eberhart, R. In *Proceedings of ICNN'95 - International Conference on Neural Networks.* 1942–1948 vol.1944.

[2] Yang, X.-S. In *Stochastic Algorithms: Foundations and Applications.* (eds Osamu Watanabe & Thomas Zeugmann) 169–178 (Springer, Berlin) (2009).

[3] Mirjalili S (2016). Dragonfly algorithm: a new meta-heuristic optimization technique for solving single-objective, discrete, and multi-objective problems. Neural Comput. Appl..

[4] Mirjalili S, Lewis A (2016). The whale optimization algorithm. Adv. Eng. Softw..

[5] Mirjalili S, Mirjalili SM, Lewis A (2014). Grey wolf optimizer. Adv. Eng. Softw..

[6] Wang G-G, Deb S, Cui Z (2019). Monarch butterfly optimization. Neural Comput. Appl..

[7] Wang G-G, Deb S, Coelho LDS (2018). Earthworm optimisation algorithm: a bio-inspired metaheuristic algorithm for global optimisation problems. Int. J. Bio-Inspired Comput..

[8] Wang, G.-G., Deb, S. & Coelho, L. D. S. In *2015 3rd International Symposium on Computational and Business Intelligence (ISCBI).* 1–5 (IEEE).

[9] Wang G-G (2018). Moth search algorithm: a bio-inspired metaheuristic algorithm for global optimization problems. Memetic Comput..

[10] Li S, Chen H, Wang M, Heidari AA, Mirjalili S (2020). Slime mould algorithm: a new method for stochastic optimization. Futur. Gener. Comput. Syst..

[11] Heidari AA (2019). Harris hawks optimization: algorithm and applications. Futur. Gener. Comput. Syst..

[12] Moghaddam, F. F., Moghaddam, R. F. & Cheriet, M. Curved space optimization: a random search based on general relativity theory. *arXiv preprint *arXiv:1208.2214 (2012).

[13] Zheng Y-J (2015). Water wave optimization: a new nature-inspired metaheuristic. Comput. Oper. Res..

[14] Ahmadianfar I, Heidari AA, Gandomi AH, Chu X, Chen H (2021). RUN beyond the metaphor: an efficient optimization algorithm based on Runge Kutta method. Expert Syst. Appl..

[15] Rao RV, Savsani VJ, Vakharia D (2011). Teaching–learning-based optimization: a novel method for constrained mechanical design optimization problems. Comput. Aided Des..

[16] Ahmadi S-A (2017). Human behavior-based optimization: a novel metaheuristic approach to solve complex optimization problems. Neural Comput. Appl..

[17] Goldberg, D. E. Genetic algorithms in search. *Optimization, and MachineLearning* (1989).

[18] Juste K, Kita H, Tanaka E, Hasegawa J (1999). An evolutionary programming solution to the unit commitment problem. IEEE Trans. Power Syst..

[19] Holland JH (1962). Outline for a logical theory of adaptive systems. J. ACM.

[20] Patro, S. P., Nayak, G. S. & Padhy, N. Heart disease prediction by using novel optimization algorithm: a supervised learning prospective. *Inform. Med. Unlocked* **26**, 100696, doi:10.1016/j.imu.2021.100696 (2021)

[21] Li X, Sun Y (2020). Stock intelligent investment strategy based on support vector machine parameter optimization algorithm. Neural Comput. Appl..

[22] Sang-To T (2022). Combination of intermittent search strategy and an improve particle swarm optimization algorithm (IPSO) for model updating. Frattura ed Integrità Strutturale.

[23] Minh, H.-L. *et al.* In *Proceedings of the 2nd International Conference on Structural Damage Modelling and Assessment.* 13–26 (Springer).

[24] Yao, X., Liu, Y. & Lin, G. Evolutionary programming made faster. *IEEE Trans. Evol. Comput.* **3**, 82–102, doi:10.1109/4235.771163 (1999).

[25] Digalakis JG, Margaritis KG (2001). On benchmarking functions for genetic algorithms. Int. J. Comput. Math..

[26] Yang, X.-S. Test problems in optimization. *arXiv preprint *arXiv:1008.0549 (2010).

[27] Rashedi E, Nezamabadi-Pour H, Saryazdi S (2009). GSA: a gravitational search algorithm. Inf. Sci..

[28] Zhao W, Wang L, Zhang Z (2019). Atom search optimization and its application to solve a hydrogeologic parameter estimation problem. Knowl.-Based Syst..

[29] Yang Y, Chen H, Heidari AA, Gandomi AH (2021). Hunger games search: Visions, conception, implementation, deep analysis, perspectives, and towards performance shifts. Expert Syst. Appl..

[30] Wu, G., R. Mallipeddi, and P. N. Suganthan. Problem definitions and evaluation criteria for the CEC 2017 competition on constrained real-parameter optimization. (researchgate, 2017).

[31] Price, K., Awad, N., Ali, M. & Suganthan, P. The 100-digit challenge: problem definitions and evaluation criteria for the 100-digit challenge special session and competition on single objective numerical optimization. *Nanyang Technol. Univ.* (2018).

[32] Abualigah L, Diabat A, Mirjalili S, Abd Elaziz M, Gandomi AH (2021). The arithmetic optimization algorithm. Comput. Methods Appl. Mech. Eng..

[33] He Q, Wang L (2007). An effective co-evolutionary particle swarm optimization for constrained engineering design problems. Eng. Appl. Artif. Intell..

[34] Mezura-Montes E, Coello CAC (2008). An empirical study about the usefulness of evolution strategies to solve constrained optimization problems. Int. J. Gen Syst.

[35] Coello CAC (2000). Use of a self-adaptive penalty approach for engineering optimization problems. Comput. Ind..

[36] Mahdavi M, Fesanghary M, Damangir E (2007). An improved harmony search algorithm for solving optimization problems. Appl. Math. Comput..

[37] Belegundu, A. D. & Arora, J. S. A study of mathematical programming methods for structural optimization. Part I: theory. *Int. J. Numer. Methods Eng.* **21**, 1583–1599, doi:10.1002/nme.1620210904 (1985).

[38] Arora, J. Introduction to optimum design with MATLAB. *Introduction to Optimum Design*, 413–432 (2004).

[39] Huang F-Z, Wang L, He Q (2007). An effective co-evolutionary differential evolution for constrained optimization. Appl. Math. Comput..

[40] Lee KS, Geem ZW (2005). A new meta-heuristic algorithm for continuous engineering optimization: harmony search theory and practice. Comput. Methods Appl. Mech. Eng..

[41] Ragsdell KM, Phillips DT (1976). Optimal design of a class of welded structures using geometric programming. J. Eng. Ind..

[42] Deb K (1991). Optimal design of a welded beam via genetic algorithms. AIAA J..

[43] Deb K (2000). An efficient constraint handling method for genetic algorithms. Comput. Methods Appl Mech Eng.

[44] Coello Coello CA (2000). Constraint-handling using an evolutionary multiobjective optimization technique. Civ. Eng. Syst..

[45] Coello CAC, Montes EM (2002). Constraint-handling in genetic algorithms through the use of dominance-based tournament selection. Adv. Eng. Inform..

[46] Deb, K. In *Evolutionary Algorithms in Engineering Applications* 497–514 (Springer, 1997).

[47] Kaveh, A. & Talatahari, S. An improved ant colony optimization for constrained engineering design problems. *Eng. Comput.* (2010).

[48] Mirjalili S (2015). Moth-flame optimization algorithm: a novel nature-inspired heuristic paradigm. Knowl.-Based Syst..

[49] Kannan BK, Kramer SN (1994). An augmented lagrange multiplier based method for mixed integer discrete continuous optimization and its applications to mechanical design. J. Mech. Des..

[50] Sandgren E (1990). Nonlinear integer and discrete programming in mechanical design optimization. J. Mech. Des..

